# Bile Salt Hydrolases with Extended Substrate Specificity Confer a High Level of Resistance to Bile Toxicity on Atopobiaceae Bacteria

**DOI:** 10.3390/ijms231810980

**Published:** 2022-09-19

**Authors:** Kana Morinaga, Hiroyuki Kusada, Hideyuki Tamaki

**Affiliations:** 1 Bioproduction Research Institute, National Institute of Advanced Industrial Science and Technology, Tsukuba 305-8566, Ibaraki, Japan; 2 Faculty of Life and Environmental Sciences, University of Tsukuba, Tsukuba 305-8572, Ibaraki, Japan

**Keywords:** bile resistance, bile salt hydrolase, *Granulimonas faecalis*, *Leptogranulimonas caecicola*, probiotics

## Abstract

The bile resistance of intestinal bacteria is among the key factors responsible for their successful colonization of and survival in the mammalian gastrointestinal tract. In this study, we demonstrated that lactate-producing Atopobiaceae bacteria (*Leptogranulimonas caecicola* TOC12^T^ and *Granulimonas faecalis* OPF53^T^) isolated from mouse intestine showed high resistance to mammalian bile extracts, due to significant bile salt hydrolase (BSH) activity. We further succeeded in isolating BSH proteins (designated LcBSH and GfBSH) from *L. caecicola* TOC12^T^ and *G. faecalis* OPF53^T^, respectively, and characterized their enzymatic features. Interestingly, recombinant LcBSH and GfBSH proteins exhibited BSH activity against 12 conjugated bile salts, indicating that LcBSH and GfBSH have much broader substrate specificity than the previously identified BSHs from lactic acid bacteria, which are generally known to hydrolyze six bile salt isomers. Phylogenetic analysis showed that LcBSH and GfBSH had no affinities with any known BSH subgroup and constituted a new BSH subgroup in the phylogeny. In summary, we discovered functional BSHs with broad substrate specificity from Atopobiaceae bacteria and demonstrated that these BSH enzymes confer bile resistance to *L. caecicola* TOC12^T^ and *G. faecalis* OPF53^T^.

## 1. Introduction

Probiotics are defined as “live microbial feed supplements which beneficially affect the host animal by improving its intestinal microbial balance” [1]. A diverse array of probiotics, especially lactic acid bacteria, have been reported to confer beneficial effects on human health [2], such as improvement in the health of individuals who are overweight [3], the alleviation of diarrhea [4], treatment for lactose intolerance [5], treatment for dyslipidemia [6], and relief from constipation [7]. Among the criteria for selecting probiotics, candidate strains are first evaluated for resistance against several stress conditions, including salivary/gastric enzymes, low pH, gastric juice, and bile [8]. In particular, bile resistance is considered essential for the survival and retention of the activity of probiotic bacteria in the mammalian gastrointestinal tract [8]. As bile acids are strong surfactants that are ubiquitously present in gut ecosystems, bile exposure in the gastrointestinal tract is highly toxic to gut bacteria. Previous studies have reported that bile concentrations of 0.3% to 2.0% are considered critical for the selection of probiotic candidates [8,9,10].

Bile resistance is primarily mediated by bile salt hydrolase (BSH) enzymes (EC3.5.1.24), which deconjugate glycine and/or taurine-conjugated bile acids [11,12,13], thereby leading to the detoxification of conjugated bile acids, although alternative stress tolerance mechanisms (e.g., efflux transporter and cell wall modification [14]) might also be involved in bile resistance. BSH enzymes have been known to confer further beneficial effects on human health, including antimicrobial [15,16,17] and cholesterol-lowering activities [12]. In general, BSH enzymes have been identified in probiotic candidates within the phylum Firmicutes (i.e., genera *Clostridium* [18], *Christensenella* [19], *Enterococcus* [20], *Lacticaseibacillus* [21], *Lactiplantibacillus* [22], *Lactobacillus* [23,24,25], *Ligilactobacillus* [26], *Listeria* [27], and *Lysinibacillus* [28]). Although gut bacteria belonging to the phylum Actinobacteria have also been widely recognized as beneficial probiotic candidates [29], previous research related to BSH has been limited to members of the genus *Bifidobacterium* within the family Bifidobacteriaceae [30,31,32,33,34].

Members of the Atopobiaceae family (phylum Actinobacteria) have recently been recognized as potentially beneficial microbes in the mammalian gastrointestinal tract [35]. Ducarmon et al. reported that gut microbes within the Atopobiaceae family may be associated with protection against multidrug-resistant bacterial colonization in elderly individuals [36]. Furthermore, most Atopobiaceae bacteria have been reported to produce beneficial lactate and short-chain fatty acids, including acetate and butyrate, as fermentation products [37,38,39]. Selle et al. demonstrated that the relative abundance of operational taxonomic units related to the Atopobiaceae family significantly increased after prebiotic (galacto-oligosaccharides/inulin) supplementation in a mouse gut model [40]. Despite these probiotic potentials, Atopobiaceae bacteria have not yet been used practically, due to the lack of a genetic/enzymatic characterization of their probiotic properties. Some Atopobiaceae bacteria have been reported to exhibit high bile resistance ability [41,42]; however, their bile resistance mechanism has not yet been elucidated.

To elucidate the correlation between bile resistance and BSH enzymes in gut Atopobiaceae bacteria, we investigated whether two novel probiotic candidates within the Atopobiaceae family, *Leptogranulimonas caecicola* TOC12^T^ and *Granulimonas faecalis* OPF53^T^, represent bile resistance ability in different mammalian bile extracts. We previously demonstrated that *L. caecicola* TOC12^T^ and *G. faecalis* OPF53^T^ were isolated from mouse intestines and produced relatively high level of lactate as the predominant fermentation product [43]. In general, lactate-producing bacteria have been recognized as beneficial microorganisms and are widely used as probiotic agents to maintain host health by reducing gut pH and, thereby, growth inhibition of gut pathogens [44,45,46]. Furthermore, we explored new gene candidates encoding BSH from *L. caecicola* TOC12^T^ and *G. faecalis* OPF53^T^ and characterized their biochemical properties and phylogeny.

## 2. Results and Discussion

### 2.1. Bile Resistance in L. caecicola TOC12^T^ and G. faecalis OPF53^T^

We first investigated whether *L. caecicola* TOC12^T^ and *G. faecalis* OPF53^T^ could resist bile extracts, using a standard plate assay. We observed that both strains were able to grow and form colonies on GAM agar plates containing 2.0% oxgall (cow bile extract), clearly indicating that *L. caecicola* TOC12^T^ and *G. faecalis* OPF53^T^ possess bile resistance. Considering that the average bile concentration in mammalian intestines has been estimated to be 0.3% *w*/*v* [47], both strains TOC12^T^ and OPF53^T^ have a high bile resistance capability. Furthermore, we determined the minimal inhibitory concentrations (MICs) of cow and porcine bile extracts against both strains. As shown in Table 1, strain TOC12^T^ showed high MIC values for both cow and porcine bile extracts (MICs of 4.0%). Interestingly, strain OPF53^T^ also displayed a 4.0% MIC value for cow bile extract; however, this organism exhibited a much lower MIC value (MIC of 0.1%) for porcine bile extract (Table 1).

We assumed that the different MIC profiles of strain OPF53^T^ could be attributed to the different bile salt compositions of cow and porcine bile extracts. Porcine bile extracts have been reported to be mainly composed of glyco/tauro-hyodeoxycholic acids (GHDCA and THDCA) and glyco/tauro-chenodeoxycholic acids (GCDCA and TCDCA) [48]. In contrast, cow bile extracts are primarily composed of glyco/tauro-cholic acids (GCA and TCA) and glyco/tauro-deoxycholic acids (GDCA and TDCA) [48]. Thus, our findings, together with a previous report by Watanabe et al. [48], suggested that strain TOC12T resists various bile salts, including GCA, TCA, GDCA, TDCA, GHDCA, THDCA, GCDCA, and TCDCA, whereas strain OPF53^T^ may be susceptible to porcine-specific bile salts (i.e., GHDCA, THDCA, GCDCA, and TCDCA).

**Figure 1:**
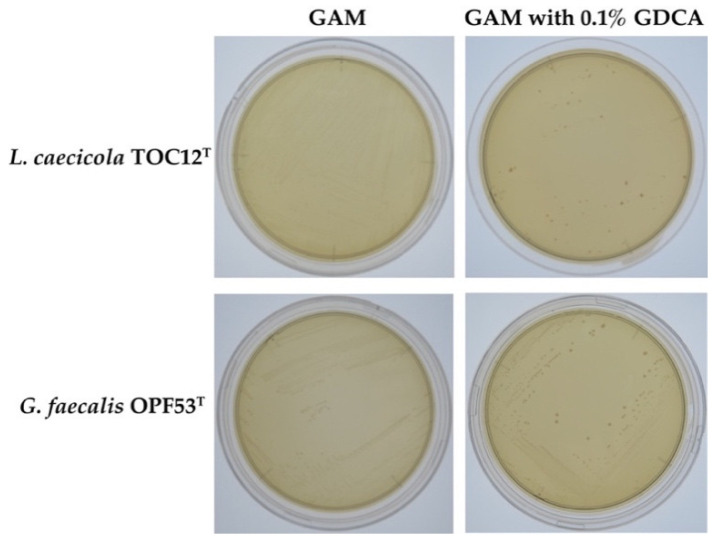
Bile salt hydrolase (BSH) activity in *Leptogranulimonas caecicola* TOC12^T^ and *Granulimonas faecalis* OPF53^T^. Full-grown cultures were streaked on a Gifu anaerobic medium (GAM) agar plate (**left**) and a GAM agar plate supplemented with 0.1% glycodeoxycholic acid (**right**). All plates were anaerobically incubated at 37 °C for 5 days. The visible precipitates surrounding colonies are the well-known indicators of bacterial BSH activity [12,27].

To verify the bile resistance mechanism of strains TOC12^T^ and OPF53^T^, we examined whether both strains showed BSH activity. We observed that visible deconjugated deoxycholic acid (DCA) precipitates surrounding colonies (a well-known indicator of bacterial BSH activity [12,27]) only when both strains were cultured on GAM agar plates supplemented with glycodeoxycholic acid (GDCA), one of the major conjugated bile salts present in oxgall (Figure 1). Hence, we attributed the high bile resistance observed in strains TOC12^T^ and OPF53^T^ to their BSH activity. Interestingly, some recent studies have demonstrated that BSH-producing gut bacteria (*Lactobacillus gasseri* CNCM I-4884 and *Lactobacillus* *johnsonii* La1) show significant anti-parasitic activity against the well-known intestinal protozoan parasite *Giardia duodenalis* [15,49]. Furthermore, Yoon et al. reported that BSH-producing *Bacteroides ovatus* SNUG 40239 exhibited a strong growth inhibitory effect on *Clostridium difficile*, one of the major causes of nosocomial diarrheal diseases [17]. More importantly, the anti-*Giardia* and anti-*Clostridium* activities of gut bacteria have been reported to be correlated with their BSH-producing activities (i.e., deconjugated-DCA compounds produced by BSH activity can inhibit the growth of *G. duodenalis* and *C. difficile*). Although further investigations are required, two BSH-producing Atopobiaceae bacteria may have anti-pathogenic activities.

### 2.2. Screening and Sequence Analyses of Putative BSH Genes

**Figure 2:**
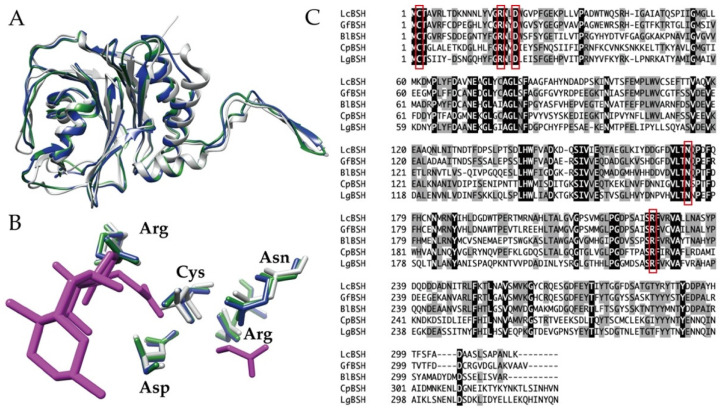
Sequence analyses of the putative LcBSH and GfBSH. (**A**) Overall and (**B**) catalytic active site superposition images of LcBSH (blue) and GfBSH (green) with CpBSH from *Clostridium perfringens* (gray; PDB accession number 2RLC). The degradation products (glycine and cholic acid) of glycocholic acid by CpBSH are shown in magenta sticks. (**C**) Multiple alignment analysis of the putative LcBSH and GfBSH. Amino acid sequences of LcBSH and GfBSH were compared with characterized bile salt hydrolases (BSHs) from gut bacteria. The black and gray backgrounds indicate identical and similar amino acid residues, respectively. The conserved residues (Cys, Arg, Asp, Asn, and Arg) related to the catalytic active site are boxed in red lines. Abbreviated as: BlBSH (AAF67801) from *Bifidobacterium longum* SBT2928; CpBSH (P54965) from *Clostridium perfringens* 13; LgBSH (WP_020806888) from *Lactobacillus gasseri* FR4.

We explored putative BSH genes from the whole genome sequences of *L. caecicola* TOC12^T^ and *G. faecalis* OPF53^T^. Based on sequence analyses, we identified putative BSH gene candidates (designated *lcBSH* and *gfBSH*) in *L. caecicola* TOC12^T^ and *G. faecalis* OPF53^T^, respectively. Signal peptides were not predicted by SignalP program. The putative *lcBSH* and *gfBSH* genes comprised 948 and 954 bp, respectively. The conserved domain search revealed that the amino acid sequences of both LcBSH (315 amino acids) and GfBSH (317 amino acids) were associated with the cholylglycine hydrolase family proteins of the Ntn-hydrolase superfamily proteins. Our three-dimensional modeling analyses further showed that the overall structures of LcBSH and GfBSH constituted the well-known αββα-sandwich folds of cholylglycine hydrolase proteins (Figure 2A), as well as structurally characterized BSH (CpBSH) from *Clostridium perfringens* 13 [50]. Multiple alignments and structural superposition analyses indicated that five amino acid residues (Cys, Arg, Asp, Asn, and Arg) responsible for the catalytic active site were highly conserved in both the LcBSH and GfBSH proteins (Figure 2B,C). Therefore, these sequence analyses strongly suggest that these putative BSHs (LcBSH and GfBSH) could serve as functional BSH enzymes.

### 2.3. Enzymatic Activity of Heterologously Expressed LcBSH and GfBSH Proteins

**Figure 3:**
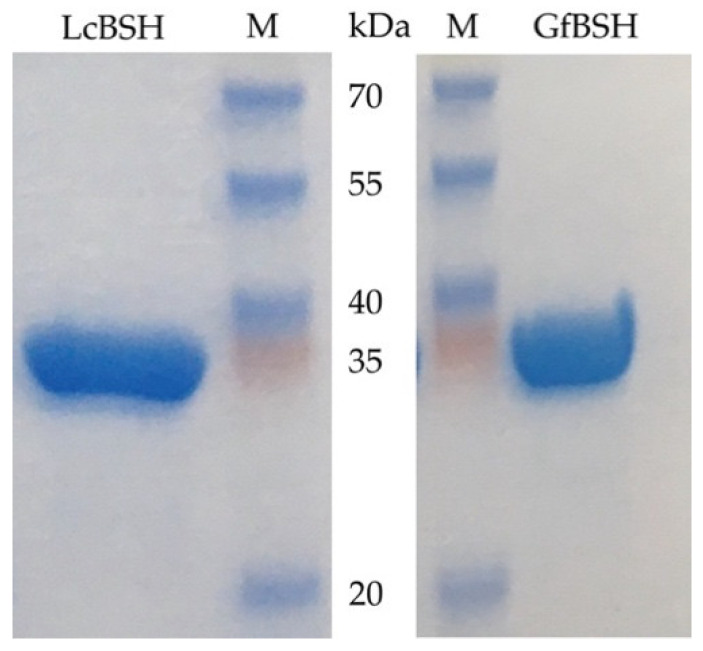
Sodium dodecyl sulfate polyacrylamide gel electrophoresis (SDS-PAGE) analysis of purified LcBSH and GfBSH. The purified proteins were loaded onto 12% SDS-PAGE gels. The single protein bands of purified His_6_-LcBSH and His_6_-GfBSH proteins were observed to be around 35 kDa. Lane M, molecular size-marker (3-color prestained XL-Ladder, APRO Science, Tokushima, Japan).

We cloned and overexpressed the putative genes encoding *lcBSH* and *gfBSH* in *Escherichia coli*. The *lcBSH* and *gfBSH* genes were commercially synthesized with codon optimization for *E. coli*, then subcloned into expression vectors (see Section 3). The recombinant LcBSH and GfBSH proteins were purified by Ni-affinity chromatography and their molecular weights were approximately 35.0 kDa, as determined by SDS-PAGE analyses (Figure 3), which were nearly identical to their theoretical molecular weights according to their amino acid sequences.

**Figure 4:**
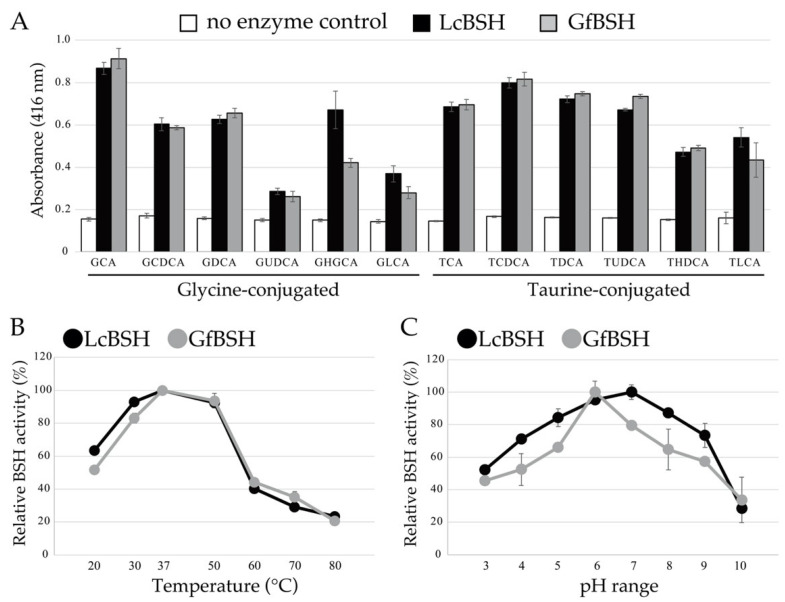
Bile salt hydrolase (BSH) activity and biochemical characterization of recombinant LcBSH and GfBSH proteins. (**A**) BSH activity and substrate specificity of LcBSH and GfBSH. The tested substrates were glycocholic acid (GCA), glycochenodeoxycholic acid (GCDCA), glycodeoxycholic acid (GDCA), glycoursodeoxycholic acid (GUDCA), glycohyodeoxycholic acid (GHDCA), glycolithocholic acid (GLCA), taurocholic acid (TCA), taurochenodeoxycholic acid (TCDCA), taurodeoxycholic acid (TDCA), tauroursodeoxycholic acid (TUDCA), taurohyodeoxycholic acid (THDCA), and taurolithocholic acid (TLCA). Values represent the mean of three independent experiments (each *n* = 3). Error bars represent standard deviation (SD). (**B**) Effect of temperature (20–80 °C) and (**C**) pH (pH 3.0–pH 10.0) on BSH activity of LcBSH and GfBSH. Each value represents the mean of six technical replicates (each *n* = 6). Maximum activity was defined as 100%. Error bars indicate SD.

To investigate the enzymatic activities of LcBSH and GfBSH, we performed a BSH activity assay and determined the substrate specificity of the recombinant proteins. As shown in Figure 4A, recombinant LcBSH and GfBSH proteins hydrolyzed all the tested conjugated bile salts, indicating that LcBSH and GfBSH are functional BSH proteins. Furthermore, LcBSH and GfBSH proteins showed similar substrate specificity and hydrolyzed both glycine-conjugated bile salts and taurine-conjugated bile salts, although previously identified BSHs from gut bacteria have been widely known to preferentially hydrolyze glycine-conjugated bile salts rather than taurine-conjugated bile salts [18,33,51]. In addition, most of the characterized BSHs from the *Lactobacillus* and *Bifidobacterium* species have been reported to hydrolyze six major conjugated bile salts (GCA, GCDCA, GDCA, TCA, TCDCA, and TDCA). However, the hydrolytic activity of known BSHs toward the remaining six minor bile salts (GUDCA, GLCA, GHDCA, TUDCA, TLCA, and THDCA) has not been fully demonstrated. With a few exceptions, BSHs from *Bifidobacterium longum* SBT2928 and *Lactobacillus paragasseri* JCM 5343^T^ have been reported to hydrolyze minor conjugated bile salts, in addition to the major ones [24,25,33]. Therefore, our findings clearly indicate that LcBSH and GfBSH are novel BSH enzymes with broad substrate specificity.

We further found that LcBSH exhibited much higher BSH activity toward GHDCA than toward GfBSH (Figure 4A), although both proteins displayed similar substrate specificity toward residual eleven substrates. The different substrate specificities of GHDCA correlated well with the MIC values of their host organisms toward bile extracts (Table 1). In fact, strain TOC12^T^ with LcBSH showed a high MIC value (4%) toward GHDCA-containing porcine bile extract, whereas strain OPF53^T^ with GfBSH displayed a much lower MIC value (0.1%) toward porcine bile extract, suggesting that the LcBSH enzyme mainly contributes to resistance to porcine bile extract in strain TOC12^T^.

### 2.4. Biochemical Characterization of LcBSH and GfBSH

We investigated the effects of temperature and pH on the enzymatic activity of LcBSH and GfBSH. The effects of temperature on the enzyme activity of the LcBSH protein were similar to those of GfBSH, and the highest BSH activities of LcBSH and GfBSH were commonly observed at 37 °C (Figure 4B). Both proteins exhibited stable activity and retained more than 80% of their original activity under mesophilic conditions (30–50 °C), whereas significant decreases in enzyme activity were observed at 20 °C and temperatures higher than 60 °C. We previously demonstrated that both strains grew well at temperatures between 30 °C and 45 °C (optimum at 37 °C and 37–40 °C for strains TOC12^T^ and OPF53^T^, respectively) [43], indicating that the optimum temperatures of LcBSH and GfBSH are closely consistent with the growth conditions of *L. caecicola* TOC12^T^ and *G. faecalis* OPF53^T^.

However, the effects of pH on enzyme activity varied considerably between LcGSH and GfBSH proteins (Figure 4C). The maximum BSH activity of LcBSH was observed at pH 7.0, whereas that of GfBSH was observed at pH 6.0. The optimum pH values of previously identified BSHs were relatively acidic (e.g., the optimum pH values of BSHs from *L. jounsonii* strains and *C. perfringens* are 3.8–4.5 and 4.5, respectively [13]). Furthermore, LcBSH was functionally stable with above 80% of its residual activity over a broad pH range (pH 5.0–8.0), although GfBSH showed pH-sensitive properties with below 80% of its residual activity at pH 3.0–5.0 and pH 7.0–10.0.

### 2.5. Sequence Comparison and Phylogenetic Analyses

A BLASTP (protein–protein BLAST) search revealed that LcBSH and GfBSH exhibited relatively low amino acid sequence similarity to characterized BSHs. Moreover, even the highest amino acid sequence similarities of LcBSH and GfBSH were only 43.97% and 50.49%, respectively, with BSH from *Bifidobacterium adolescentis* ATCC 15705 [31]. In contrast, LcBSH and GfBSH shared a higher sequence similarity (61.46% similarity), indicating that LcBSH and GfBSH are new BSHs with low sequence similarity to previously identified BSHs from gut bacteria.

**Figure 5:**
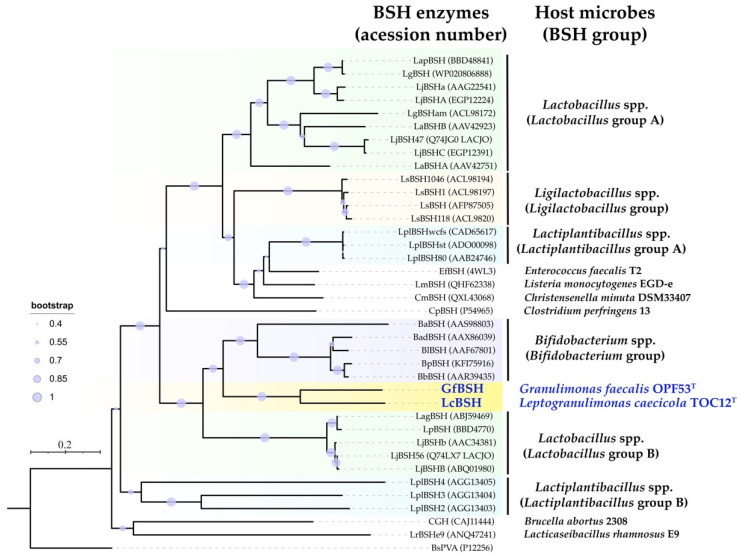
Phylogenetic analysis of LcBSH and GfBSH. Amino acid sequences of LcBSH and GfBSH proteins were aligned with previously identified bile salt hydrolases (BSHs). The phylogenetic tree was constructed with MEGA X software, using the neighbor-joining method (1000 bootstrap replications) [52]. The evolutionary distances were computed using the Poisson correction method and are in the units of the number of amino acid substitutions per site. The phylogenetic tree was displayed and customized using the online tool Interactive Tree Of Life (iTOL v6) [53]. Bootstrap values are shown by circle symbols whose size correlates with the bootstrap values. BsPVA from *Lysinibacillus sphaericus* was used as an outgroup.

We performed phylogenetic analysis using the amino acid sequences of LcBSH, GfBSH, and the characterized BSHs. As shown in Figure 5, previously isolated BSH proteins were subdivided into six major groups, based on bacterial taxonomy (i.e., *Lactobacillus* group A, *Lactobacillus* group B, *Ligilactobacillus* group, *Lactiplantibacillus* group A, *Lactiplantibacillus* group B, *Bifidobacterium* group). We found that LcBSH and GfBSH constituted a new Atopobiaceae BSH subgroup (Figure 5, yellow background), and the new BSH group was clearly distinguishable from other BSH groups with high bootstrap values, suggesting that LcBSH and GfBSH are phylogenetically novel BSHs.

To date, most characterized BSHs have been isolated from lactic acid bacteria within the phylum Firmicutes (Figure 5). As for members of the phylum Actinobacteria, BSHs have been found only in *Bifidobacterium* species. In the present study, we discovered two novel BSHs from Atopobiaceae bacteria (phylum Actinobacteria) and demonstrated their phylogenetic novelty. This result suggested that a diverse array of gut bacteria within the phylum Actinobacteria would possess BSH activity and the corresponding BSH enzyme to resist high concentrations of conjugated bile acids and, therefore, be able to colonize the mammalian digestive tract. Our extended metagenomic database search clarified that putative BSHs were broadly present in the genomes of the phylum Actinobacteria, suggesting that the yet-to-be cultured BSH-producing Actinobacteria is a potential candidate for future probiotic research.

## 3. Materials and Methods

### 3.1. Bacterial Strains and Their Culture Conditions

Two strictly anaerobic, Gram-positive, and lactate-producing bacteria, *Leptogranulimonas caecicola* TOC12^T^ (JCM 35017^T^ = KCTC 25472^T^) and *Granulimonas faecalis* OPF53^T^ (JCM 35015^T^ = KCTC 25474^T^), were previously isolated from the gastrointestinal tract of mice [43]. These strains were cultivated in a modified Gifu anaerobic medium (GAM; Nissui Pharmaceutical Co., Ltd., Tokyo, Japan) with headspace gas of N_2_/CO_2_ (80:20, *v*/*v*) at 37 °C under anaerobic conditions. *Escherichia coli* strain BL21 (DE3) Champion^TM^21 (SMOBIO Technologies, Hsinchu City, Taiwan) was used as the host strain for heterologous expression experiments. *E. coli* strains were cultured in Luria-Bertani broth (LB broth, Nacalai Tesque, Kyoto, Japan) supplemented with 50 μg/mL kanamycin (FUJIFILM Wako Pure Chemical Corporation, Osaka, Japan) or 100 μg/mL ampicillin (Sigma-Aldrich, Saint Louis, MO, USA) at 37 °C with vigorous shaking.

### 3.2. Screening and Heterologous Expression of Putative Bile Salt Hydrolase (BSH) Genes

We screened putative BSH genes from the whole genome sequences of strains TOC12^T^ (accession number AP025285) and OPF53^T^ (accession number BQKC01000001), based on homology and domain searches using the NCBI BLAST program (https://blast.ncbi.nlm.nih.gov/Blast.cgi, accessed on 8 May 2022), UniProt BLAST tool (https://www.uniprot.org/blast/, accessed on 8 May 2022), InterProScan (http://www.ebi.ac.uk/interpro/search/sequence-search, accessed on 8 May 2022), and Pfam (http://pfam.xfam.org/, accessed on 8 May 2022). The presence of signal peptides was predicted using the SignalP-6.0 server (https://services.healthtech.dtu.dk/service.php?SignalP, accessed on 8 May 2022). Putative *bsh* genes from strains TOC12^T^ and OPF53^T^ (named *lcBSH* and *gfBSH*, respectively) were commercially synthesized with codon optimization for heterologous expression in *E. coli* (Genscript, Piscataway, NJ, USA) (see Supplementary Materials). The *lcBSH* and *gfBSH* genes were cloned into the *Nde*I and *Eco*RI sites of the pET28b (kanamycin resistance; Novagen, Madison, WI, USA) and pColdII (ampicillin resistance; TaKaRa, Tokyo, Japan) expression vectors, respectively. We designed the His_6_-tags to be attached to the N-terminus of the recombinant LcBSH and GfBSH proteins.

We performed heterologous gene expression and protein purification experiments, as described in our previous studies [23,24,25,54]. In brief, the constructed plasmids (pET28b-*lcBSH* and pColdII-*gfBSH*) were transformed into competent *E. coli* BL21 (DE3) Champion^TM^21 cells. *E. coli* strains were cultured in LB broth at 37 °C until the optical density (OD_600_) reached approximately 0.5. Isopropyl-β-D-thiogalactopyranoside (IPTG; Nacalai Tesque) was added to the culture media at a final concentration of 100 μM. *E. coli* cells were further incubated at 20 °C or 15 °C overnight with shaking. The cells were centrifuged at 5800× *g* for 10 min and suspended in lysis buffer (20 mM Tris, 150 mM NaCl, 5% glycerol, and 5 mM imidazole, pH 7.0). The cells were disrupted by sonication using an ultrasonic disintegrator (Sonicator BRANSON Sonifer 250, Branson, Danbury, CT, USA; output control:5, duty cycle:50) in an ice-water bath (five times for 1 min each). After sonication, cell-free soluble protein fractions were purified by Ni-affinity chromatography on Ni-NTA agarose HP (FUJIFILM Wako Pure Chemical Corporation). To remove imidazole, the purified protein solutions were dialyzed with buffer (20 mM Tris, 150 mM NaCl, and 5% glycerol) using a semipermeable membrane (Spectra/Por 3 membrane MWCO:3500, Repligen, Waltham, MA, USA), then concentrated using Vivaspin centrifugal concentrators (30,000 MWCO PES; Sartorius Stedim Biotech GmbH, Goettingen, Germany).

### 3.3. Sodium Dodecyl Sulfate Polyacrylamide Gel Electrophoresis (SDS-PAGE) Analysis

The purified recombinant proteins (LcBSH and GfBSH) were treated with 4× premixed sample buffer solution (Bio-Rad, Hercules, CA, USA) and heat-denatured at 95 °C for 5 min using a thermal cycler (TaKaRa). The resulting protein solutions were analyzed by sodium dodecyl sulfate polyacrylamide gel electrophoresis (SDS-PAGE) using Mini-PROTEAN TGX precast polyacrylamide gels (Bio-Rad), according to the methods described in previous studies [23,24,25,54]. The gels were stained overnight with QC Colloidal Coomassie Stain (Bio-Rad), with gentle agitation, then decolorized with deionized water.

### 3.4. Enzymatic Activity

The bile salt hydrolase activity of the recombinant LcBSH and GfBSH proteins was assayed, as previously described [16,24,25,55]. Purified proteins were mixed with 0.24 mg/100 μL of conjugated bile salt solutions and incubated at 37 °C. In addition, each bile salt solution was mixed with a buffer solution (20 mM Tris, 150 mM NaCl, and 5% glycerol) instead of purified proteins and used as a negative control. The enzymatic reactions were terminated by the addition of 15% trichloroacetic acid (FUJIFILM Wako Pure Chemical Corporation). The denatured proteins were removed by centrifugation at 10,000× *g* for 15 min at 20 °C. The supernatant was then reacted with 300 mM borate buffer containing 1% SDS (pH 9.5) and 0.3% 2,4,6-trinitrobenzenesulfonic acid solution (Tokyo Kasei Kogyo Co., Ltd., Tokyo, Japan). The reaction mixtures were statically incubated for 30 min at room temperature under dark conditions; finally, 0.6 mM HCl was added to stop the reaction. Absorbance at 416 nm was measured using a SPARK 10M multimode microplate reader (TECAN, Männedorf, Switzerland). The assays were performed in triplicate. The Student’s *t*-test was used to assess the presence of statistically significant differences using GraphPad Prism software (version 8.0; GraphPad Software, San Diego, CA, USA).

### 3.5. Biochemical Characterization

The effects of temperature and pH on the enzymatic activity of the LcBSH and GfBSH proteins were evaluated, as previously described [24,25,55]. The purified proteins were mixed with taurodeoxycholic acid (TDCA) at selected ranges of temperature (20 °C to 80 °C, in intervals of 10 °C) and pH (pH 3.0 to pH 10.0, in intervals of pH 1.0). After incubation for 6 h, released taurine was detected, as described above. All experiments were performed in six technical replicates. The following Good’s buffer solutions were used to adjust the pH values by replacing the buffer in the reaction mixture according to a previous study [55]: acetate buffer (CH_3_COONa∙3H_2_O) for pH 3.0–4.0; MES buffer (C_6_H_13_NO_4_S∙H_2_O) for pH 5.0–6.0; HEPES buffer (C_8_H_18_N_2_O_4_S) for pH 7.0–8.0; CAPS buffer (C_9_H_19_NO_3_S) for pH 9.0–10.0.

### 3.6. Bile Resistance Tests

The bile resistance of *L. caecicola* TOC12^T^ and *G. faecalis* OPF53^T^ was assayed, as previously described [9,56]. Strains TOC12^T^ and OPF53^T^ were cultivated in GAM broth and inoculated on GAM agar plates containing 2.0% oxgall (Difco Laboratories, Detroit, MI, USA). Plates were anaerobically incubated at 37 °C for 4 days using an AnaeroPack system with an oxygen absorber (Mitsubishi Gas Chemical America, Inc., Tokyo, Japan). Minimum inhibitory concentrations (MICs) were determined as the lowest concentration of bile extracts that prevented visible growth of *L. caecicola* TOC12^T^ and *G. faecalis* OPF53^T^ on GAM agar plates. Oxgall and porcine bile extract (Sigma-Aldrich) were used as substrates. The final concentrations of the bile extracts were 0.05%, 0.1%, 1.0%, 2.0%, 4.0%, and 6.0%. All experiments were performed in triplicate.

### 3.7. Structural Modeling, Multiple Alignment, and Phylogenetic Analyses

Three-dimensional modeling analyses of LcBSH and GfBSH were performed using the Swiss-Model workspace (https://swissmodel.expasy.org/, accessed on 8 May 2022) [57]. A BSH from *Bifidobacterium longum* [58] (PDB ID:2HF0) was used as a template for structural modeling. Superposition modeling analyses were performed and visualized using UCSF Chimera software [59]. Structurally characterized BSH from *Clostridium perfringens* (PDB ID:2RLC) was obtained from the Protein Data Bank (http://www.rcsb.org/pdb/home/home.do, accessed on 8 May 2022). Multiple amino acid sequence alignment analysis was performed using GENETYX-MAC software version 20.1.1 (GENETYX, Tokyo, Japan) and the CLUSTAL W2 program. In addition, a neighbor-joining phylogenetic tree was constructed with 1000 bootstrap replications using MEGA X software [52] and visualized using the online tool Interactive Tree Of Life (iTOL v6) (https://itol.embl.de/, accessed on 8 May 2022) [53].

## 4. Conclusions

In this study, we discovered that two Atopobiaceae intestinal bacteria, *L. caecicola* TOC12^T^ and *G. faecalis* OPF53^T^, showed high resistance to bile toxicity, due to significant BSH activity. Although some Atopobiaceae bacteria have been reported to show bile resistance ability, we demonstrated for the first time that BSH enzymes critically mediate the high resistance to bile extracts in Atopobiaceae bacteria by isolating and characterizing their functional BSH enzymes (designated as LcBSH and GfBSH). We assumed that these BSH enzymes would provide survival advantages for strains TOC12^T^ and OPF53^T^ to resist bile toxicity and thereby colonize the host digestive tract. BSH activity has been reported to confer further ecological advantages on human health, such as pathogen killing, weight reduction, and cholesterol-lowering activities [12]; therefore, further study is required to verify the in vivo probiotic effects of BSH-producing Atopobiaceae species using mouse model experiments.

The present study further demonstrated the enzymatic and phylogenetic uniqueness of LcBSH and GfBSH proteins. Both BSH proteins showed functional BSH activity toward 12 different conjugated bile salts, clearly indicating that these new BSHs have broader substrate specificity than the well-known BSHs from probiotic *Lactobacillus* and *Bifidobacterium* species. Phylogenetic analysis further clarified that the LcBSH and GfBSH proteins were affiliated with distinct BSH phylogroups which have low amino acid sequence similarity to previously identified BSHs.

Overall, this study expands the current understanding of the phylogenetic diversity of BHS-producing probiotic candidates in mammalian digestive tract. Furthermore, our findings suggest that BSH-producing Atopobiaceae bacteria and their BSH enzymes with broad substrate specificity could be regarded as new targets for future probiotic research.

## Figures and Tables

**Table 1:** Minimum inhibitory concentrations of bile extracts against strains TOC12^T^ and OPF53^T^.

Strain	Minimum Inhibitory Concentrations (%)	
	Bile Extracts	
	Cow Bile (Difco)	Porcine Bile (Sigma)
*L. caecicola* TOC12^T^	4.0	4.0
*G. faecalis* OPF53^T^	4.0	0.1

## Data Availability

Not applicable.

## Supplementary Materials

The following supporting information can be downloaded at: https://www.mdpi.com/article/10.3390/ijms231810980/s1.
