# Supplementary material for: Bile Salt Hydrolases with Extended Substrate Specificity Confer a High Level of Resistance to Bile Toxicity on Atopobiaceae Bacteria
Source: Int J Mol Sci. 2022 Sep 19;23(18):10980. doi: 10.3390/ijms231810980 (PMC9506489; doi:10.3390/ijms231810980)
Supplement: Supplementary file 1 [file ijms-23-10980-s001.zip › ijms-1909494-supplementary.pdf]

International Journal of
Molecular Sciences

MDPI

Supplementary Materials

Bile Salt Hydrolases with Extended Substrate Specificity Confer a High Level of Resistance to Bile Toxicity on Atopobiaceae Bacteria

Kana Morinaga [1,†], Hiroyuki Kusada [1,*,†] and Hideyuki Tamaki [1,2,*]

1 Bioproduction Research Institute, National Institute of Advanced Industrial Science and Technology, Tsukuba 305-8566, Ibaraki, Japan
2 Faculty of Life and Environmental Sciences, University of Tsukuba, Tsukuba 305-8572, Ibaraki, Japan
* Correspondence: kusada-hiroyuki@aist.go.jp (H.K.); tamaki-hideyuki@aist.go.jp (H.T.); Tel.: +81-29-861-6591 (H.K. & H.T.); Fax: +81-29-861-6587 (H.K. & H.T.)
† These authors contributed equally to this work

>Original sequence (lcBSH)
ATGTGCACCGCCGTTCGCTTAACCGATAAAAACAACAACCTCTATGTAGGCCGC
AATTTGGACTGGGGCGTTCCTTTTGGCGAGAAGCCTCTGCTGGTGCCTGCAGATT
GGACCTGGCAAAGCCGCCACATAGGGGCTATTGCCACTCAGTCGCCCATCATTG
GTATGGGGCTGCTCATGAAGGACATGCCTCTTTATTTTGACGCCGTGAACGAGGC
GGGCCTTTACTGCGCCGGCCTTTCCTTTGCCGCAGGCTTCGCACATTACAACGATG
CTGATCCTTCCAAGATCAACGTCACCAGTTTCGAGATGCCCCTTTGGGTATGCTCC
GAGTTCACTACAGTTGCCCAGGTGAAGGAAGCTGCTCAGAACCTCAATATCACC
AACGACACCTTCGATCCTTCGCTTCCTACTTCCGACCTGCATTGGTTCGTCGCCGA
CAAAGACCAGTCCATTGTCATCGAGCAAACCGCTGAAGGTCTTAAGATCTACGA
TGACGGCTTTGACGTGTTGACCAATCAGCCGGACTTCCAGTTCCACTGCAACAAC
ATGCGCAATTACATTCACCTGGACGGCGACTGGACACCCGAGCGCACCATGCGC
AACGCTCATCTCACCGCCCTGGGCGTAGGTCCCTCCGTGATGGGTCTTCCAGGCG
ACCCCTCTGCCATCTCGCGTTTCGTTCGCGTTGCACTGCTTAATGCAAGCTATCCC
GACCAAGACGATGACGCTGACAACATCACCCGCCTCTTCAAGACCCTCAATGCA
GTCTCTATGGTCAAGGGTTACTGCCGTCAGGAGTCTGGCGACTTTGAATACACCA
TTTACACCGGCGGTTTCGATAGCGCTACTGGCACTTATCGCTACACCACCTACGA
TGACCCTGCCTATCACACCTTCTCGTTTGCCGACGCTGCTTCTCTCTCTGCCCCAG
CCAACCTCAAGTAG

>Codon optimized sequence (lcBSH)
ATGTGCACCGCGGTTCGTCTGACCGATAAAAATAACAATCTGTATGTGGGCCGTA
ATCTGGACTGGGGCGTTCCGTTTGGCGAGAAGCCGCTGCTGGTTCCGGCGGATTG
GACCTGGCAAAGCCGTCACATTGGTGCGATCGCGACCCAAAGCCCGATTATCGG
CATGGGTCTGCTGATGAAAGACATGCCGCTGTACTTCGATGCGGTTAACGAAGCG
GGTCTGTATTGCGCGGGTCTGAGCTTTGCGGCGGGTTTCGCGCACTATAACGATG
CGGACCCGAGCAAGATCAACGTGACCAGCTTCGAAATGCCGCTGTGGGTTTGCA
GCGAGTTTACCACCGTTGCGCAAGTGAAAGAAGCGGCGCAGAACCTGAACATCA
CCAACGACACCTTCGATCCGAGCCTGCCGACCAGCGACCTGCACTGGTTTGTGGC
GGATAAAGACCAGAGCATCGTGATCGAGCAAACCGCGGAGGGTCTGAAGATCT

ATGATGACGGTTTTGACGTTCTGACCAACCAACCGGACTTCCAGTTTCACTGCAA
CAACATGCGTAACTACATCCACCTGGACGGTGATTGGACCCCGGAACGTACCAT
GCGTAACGCGCACCTGACCGCGCTGGGTGTTGGCCCGAGCGTTATGGGTCTGCCG
GGTGACCCGAGCGCGATCAGCCGTTTCGTTCGTGTGGCGCTGCTGAACGCGAGCT
ACCCGGACCAGGATGACGATGCGGACAACATCACCCGTCTGTTCAAGACCCTGA
ACGCGGTTAGCATGGTGAAGGGTTACTGCCGTCAGGAAAGCGGTGATTTCGAGT
ACACCATCTACACCGGCGGTTTTGATAGCGCGACCGGTACCTATCGTTACACCAC
CTATGACGATCCGGCGTATCATACCTTTAGCTTTGCGGATGCGGCGAGCCTGAGC
GCGCCGGCGAACCTGAAATAA

>Original sequence (gfBSH)
ATGTGCACAGCGGTCCGGTTCTGCGACCCCGAGGGTCATCTGTACTGCGGTCGTA
ACTACGACTGGGGCGTCAGCTACGGCGAGGGCCCTGTGGCCGTCCCCGCCGGCT
GGGAGTGGCGGTCGCGCCACGAGGGCACGTTCAAGACGCGCACCGGCCTGATCG
GCATGTCCATCGTCGAGGAGGGCATGCCCCTGTTCTTCGACTGCGCCAACGAGGA
CGGCCTCTATTGTGCCGGGCTCTCGTTCGCGGGTGGCTTCGGCGTCTACCGCGAC
CCCGAGGAGGGCAAGACCAACATCGCCAGCTTCGAGATGCCGCTCTGGGTGTGC
GGGACGTTCTCCTCGGTCGACGAGGTCGAGGAGGCCCTCGCCGATGCCGCGATC
ACCAACGACAGCTTCTCCTCGGCCCTCGAGCCCTCTTCCCTCCACTGGTTCGTCGC
CGACGCCGAGCGCTCCATCGTCGTCGAGCAGGACGCCGACGGCCTCAAGGTGAG
CCACGACGGCTTCGACGTCCTCACCAACCAGCCCGAGTTCAGGTTCCACTGCGA
GAACATGCGCAACTACGTGCACCTCGACAACGCCTGGACCCCCGAGGTCACCCT
GCGCGAGGAGCACCTCACCGCCATGGGCGTCGGTCCCTCGATGATGGGCCTGCC
TGGCGACCCCTCCGCCATCTCGCGCTTCGTCTGCGTCGCCATCCTCAACGCCCTCT
ATCCCGACGAGGAGGGGGAGAAGGCCAACGTCGCGCGCCTCTTCAGGACCCTCG
GGGCCGTCTCGATGGTCAAGGGCCACTGTCGCCAGGAGTCTGGCGACTTCGAGT
ACACCTTCTACACGGGGGGCTACTCCTCCGCCAGCAAGACGTATTACTACAGCA
CCTACGAGGACCCGGCTCTGCGCACCGTCACCTTCGACGACTGCAGGGGGGTCG
ACGGCCTCGCCAAGGTGGCGGCCGTCTAG

>Codon optimized sequence (gfBSH)
ATGTGCACCGCGGTTCGTTTTTGCGATCCGGAGGGTCACCTGTATTGCGGTCGTA
ACTATGATTGGGGCGTGAGCTATGGCGAGGGCCCGGTGGCGGTTCCGGCGGGTT
GGGAATGGCGTAGCCGTCACGAAGGTACCTTCAAGACCCGTACCGGTCTGATCG
GTATGAGCATCGTGGAAGAGGGTATGCCGCTGTTTTTCGACTGCGCGAACGAAG
ACGGTCTGTACTGCGCGGGCCTGAGCTTTGCGGGTGGCTTTGGTGTTTACCGTGAC
CCGGAAGAGGGCAAAACCAACATTGCGAGCTTCGAGATGCCGCTGTGGGTTTGC
GGTACCTTTAGCAGCGTTGACGAGGTTGAGGAAGCGCTGGCGGATGCGGCGATC
ACCAACGACAGCTTCAGCAGCGCGCTGGAACCGAGCAGCCTGCACTGGTTTGTT
GCGGATGCGGAACGTAGCATCGTTGTGGAACAGGACGCGGATGGTCTGAAAGTG
AGCCACGACGGTTTTGACGTTCTGACCAACCAACCGGAGTTTCGTTTCCACTGCG
AGAACATGCGTAACTACGTTCACCTGGATAACGCGTGGACCCCGGAAGTTACCC
TGCGTGAGGAACACCTGACCGCGATGGGTGTGGGTCCGAGCATGATGGGCCTGC
CGGGTGACCCGAGCGCGATCAGCCGTTTTGTGTGCGTTGCGATTCTGAACGCGCT
GTACCCGGACGAAGAAGGCGAGAAGGCGAACGTTGCGCGTCTGTTCCGTACCCT
GGGTGCGGTTAGCATGGTTAAAGGTCACTGCCGTCAAGAAAGCGGTGATTTCGA

GTACACCTTCTACACCGGCGGTTACAGCAGCGCGAGCAAAACCTACTATTACAG
CACCTACGAAGACCCGGCGCTGCGTACCGTGACCTTTGACGACTGCCGTGGCGT
GGATGGCCTGGCGAAAGTGGCGGCGGTGTAA
